# Campimetry and visual changes after RHZE treatment for tuberculosis

**DOI:** 10.1186/s40942-022-00367-3

**Published:** 2022-03-07

**Authors:** Brunella Maria Pavan Taffner, Luiz Guilherme Ito da Cruz, Flávio de Ávila Fowler, Carolynne Cardoso Nawa, Marcia Telma Savioli, Denise Silva Rodrigues, Octaviano Magalhães Junior, Rubens Belfort Junior

**Affiliations:** 1 Departament of Ophthalmology, Federal University of São Paulo, UNIFESP, São Paulo, SP Botucatu, 822, Vila Clementino Brazil; 2 Departament of Pneumology, Federal University of São Paulo, UNIFESP, São Paulo, Brazil; 3 Departament of Infectology, Instituto Clemente Ferreira, São Paulo, Brazil; 4 Research Department, Instituto de Visão, IPEPO, São Paulo, Brazil

**Keywords:** Retina, Campimetry, Tuberculosis, Toxicity

## Abstract

**Background:**

Tuberculosis (TB) caused by *Mycobacterium tuberculosis* has a high prevalence in Brazil (Global tuberculosis report 2020. Geneva: World Health Organization; 2020). The ethambutol-induced optic neuropathy damage is partly reversible, making its early diagnosis essential to reduce permanent visual damage.

**Purpose:**

To observe alterations in the computerized campimetry, Ishihara test and visual acuity secondary to rifampicin, isoniazid, pyrimethamine, ethambutol (RHZE) treatment.

**Methods:**

Patients undergoing treatment with RHZE at the tuberculosis service of the Federal University of São Paulo were recruited from March 2019 to December 2020. The best-corrected visual acuity (VA) measurements, Ishihara test and visual fields were performed at baseline, monthly, until 2 weeks after treatment.

**Results:**

Twenty-five patients were included. The VA decreased significantly (*P* = 0.0129) post-treatment compared to month 1. The mean deviation (MD) did not decrease significantly (*P* > 0.05); the pattern standard deviation (PSD) decreased post-treatment compared to month 1 (*P* = 0.0371). Changes in the Ishihara test increased significantly (*P* < 0.0001) in the second month.

**Conclusion:**

The VA and PSD decreased significantly after RHZE treatment. Changes in the Ishihara test were observed in the second month.

*Trial registration*: The Research Ethics Committee of Federal University of São Paulo, Paulista School of Medicine approved the study in March 2019. CAAE 04297018.4.0000.5505.

## Introduction

Tuberculosis (TB) caused by *Mycobacterium tuberculosis* has a high prevalence in Brazil [1]. Since 2009, the basic TB treatment in Brazil has included ethambutol in the regimen of rifampicin, isoniazid and pyrazinamide (RHZE) [2]. The adverse effects of ethambutol have been documented since its original use, with optic neuropathy the most severe with an incidence of 22.5 cases/1000 patients [3].

The clinical characteristics of ethambutol-induced optic neuropathy are similar to those of compressive optic neuropathy with retrobulbar neuritis in a subacute clinical course consisting of decreased contrast sensitivity, painless loss of central vision, cecocentral scotomas, and dyschromatopsia. The neuropathic damage is partly reversible, making its early diagnosis essential to reduce permanent visual damage.

The study goals were observation of changes the computerized campimetry, color test and visual acuity secondary to RHZE treatment.

## Methods

Patients undergoing treatment for TB with RHZE were recruited from the TB service of the Federal University of São Paulo from March 2019 to December 2020. Only patients treated continuously with ethambutol were included in the study.

The Research Ethics Committee of Federal University of São Paulo, Paulista School of Medicine approved the study. All participants provided written informed consent in agreement with the Ethics Committee according to the recommendations of the Declaration of Helsinki.

All patients provided a medical history that included a description of their daily habits, past medical history, medication use, and history of color blindness. After clinical evaluation, the best-corrected visual acuity (VA) and Ishihara test was measured before treatment. Humphrey visual field analyzer central 24.2 (Carl Zeiss, Oberkochen, Germany) were performed after 2 weeks of treatment according to the guidelines of the infectious diseases department. The exams were performed monthly during six months of treatment. The last exam was realized 2 weeks after treatment.

With intention to mitigate the learning effect of the visual field test was compared the results from month 1 to month 2.

Patients were included who had no history of optic neuropathy such as glaucoma and optic neuritis; congenital dyschromatosis, such as color blindness and retinal diseases; significant opacity on slit-lamp or funduscopic examination; visual abnormalities or visual field defects; anterior-segment diseases except for dry eye syndrome; drug use that could induce ocular toxicity, except for ethambutol; or ocular or central nervous system TB. All patients were older than 18 years.

### Statistical analysis

Data cleaning programs were developed to assess inconsistencies and prepare the database for further analysis. The data were analyzed using the STATA 14.0 program (StataCorp LP, College Station, TX).

Frequency tables were used for descriptive analysis. The normal distribution of the variables was assessed using the Shapiro–Wilk test. Clinical variables were compared between different time points using the Wilcoxon sign-ranked test. Associations between categorical variables were assessed using the chi-square test. Associations between outcomes and continuous variables were investigated using linear regression analysis. For all tests, *P* < 0.05 was considered significant.

## Results

Twenty-five patients were included in the study. Thirteen men and twelve women (mean age: 39.72 years; age’s range: 25–54 years), three patients were lost to follow-up (two in month 3 and one in month 2) and two died (liver cancer in month 2 and liver failure after month 3 of treatment). One patient cannot perform the visual field. All six patients eye exams were excluded. Six patients were white, seven black and twelve mixed race. Three patients presented with ganglionar TB, five pleural, and seventeen pulmonary disease focus (Table 1).

**Table 1:** Categorical variables of the sample

Parameter	No. (%)
Gender (%)	
Male	13 (52)
Female	12 (48)
Ethnicity (%)	
White	6 (24)
Black	7 (28)
Mixed race	12 (48)
Disease focus (%)	
Ganglionar	3 (12)
Pleural	5 (20)
Pulmonary	17 (68)
Total	25 (100)

The mean patient weight was 61.60 kg and height 1.68 m (Table 2).

**Table 2:** Descriptive analysis of the sample

	Mean ± SD (Median)
Age (years)	39.72 ± 14.32 (37.00)
Weight (Kg)	61.60 ± 10.35 (59.90)
Height (M)	1.68 ± 0.06 (1.70)
Dose (Mg)	18.72 ± 1.65 (18.36)

SD, standard deviation

Fifteen patients (60%) were formally actively employed, four (16%) unemployed, three (12%) were students, two (8%) were retired, and one (4%) was a housewife.

Seventeen (68%) patients had no comorbidities, two (8%) had primary arterial hypertension isolated, two (8%) pulmonary fibrosis, one (4%) was positive for human immunodeficiency virus (HIV), one (4%) had asthma, one (4%) had rheumatoid arthritis and primary arterial hypertension, one (4%) had hypothyroidism.

Two (8%) patients reported using marijuana and cocaine.

Fourteen (56%) patients were from the state of São Paulo, eight (32%) from northeastern Brazil, two (8%) from the other southeastern states, and one (4%) from southern Brazil.

The final sample consider for exams was 19 patients, 38 eyes.

Changes in the Ishihara test increased significantly (*P* < 0.0001) in the second month. About the Ishihara test, was considered the numbers of plates read correctly. The VA, the mean deviation (MD) and the pattern standard deviation (PSD) did not change significantly between months 1 and 2 (Table 3).

**Table 3:** Comparison of results at months 1 and 2 of treatment

Parameter	First month	Second month	*P* value
	Mean ± SD(median)	Mean ± SD(median)	
Visual acuity	0.04 ± 0.07 (0.00)	0.05 ± 0.11 (0.00)	0.7472
Ishihara	0.23 ± 0.74 (0.00)	0.82 ± 1.17 (0.00)	** < 0.0001**
VF MD	− 1.81 ± 2.48 (− 1.27)	− 2.14 ± 3.41 (− 1.24)	0.9860
VF PSD	2.65 ± 2.11 (2.09)	2.78 ± 2.15 (2.10)	1.0000

SD, standard deviation; VF MD, visual field mean deviation; VF PSD, visual field pattern standard deviation

The VA decreased significantly (*P* = 0.0129) 2 weeks after treatment compared to baseline. The mean deviation did not change significantly (P > 0.05); however, the PSD decreased after treatment compared to baseline (*P* = 0.0371) (Table 4).

**Table 4:** Comparison of results at baseline and post-treatment

Parameter	Baseline	Post- Treatment	*P* Value
	Mean ± SD(median)	Mean ± SD(median)	
Visual acuity	0.04 ± 0.06 (0.00)	0.08 ± 0.17 (0.00)	**0.0129**
Ishihara	0.41 ± 0.92 (0.00)	0.76 ± 1.18 (0.00)	0.1121
VF MD	− 1.84 ± 2.75 (− 1.22)	− 2.19 ± 5.19 (− 0.49)	0.8575
VF PSD	2.77 ± 2.38 (2.09)	2.44 ± 1.88 (1.76)	**0.0378**

SD, standard deviation; VF MD, visual field mean deviation; VF PSD, visual field pattern standard deviation

All 38 eyes included in the visual field analysis had no significant scotomas (considered more than one point < 0.5% at pattern deviation; reduced MD or PSD) at baseline (2 weeks of treatment). The Humphrey visual field analysis showed scotoma in ten eyes (26%) central; seven eyes (18%) upper arched; seven eyes (18%) upper nasal peripheral; two eyes (5%) central and upper arched; two eyes (5%)upper temporal peripheral; two eyes (5%) lower temporal peripheral; eight eyes (21%) no scotoma after RHZE treatment (Table 5).

**Table 5:** Humphrey visual field analysis 24.2 after treatment

Patient	Right eye	Left eye
1	Central scotoma	Central scotoma
2	Upper arched scotoma	Upper arched scotoma
3	No scotoma	No scotoma
4	No scotoma	Central scotoma
5	Central scotoma	Central scotoma and upper arched scotoma
6	No scotoma	No scotoma
7	Upper nasal peripheral scotoma	Upper temporal peripheral scotoma
8	Central scotoma	Central scotoma
9	Upper nasal peripheral scotoma	Lower temporal peripheral scotoma
10	Upper nasal peripheral scotoma	No scotoma
11	Central scotoma	Upper nasal peripheral scotoma
12	No scotoma	Central scotoma
13	Central scotoma	Central scotoma
14	Upper arched scotoma	Upper arched scotoma
15	Upper arched scotoma	Central scotoma and upper arched scotoma
16	No scotoma	Lower temporal peripheral scotoma
17	Upper arched scotoma	Upper nasal peripheral scotoma
18	Upper temporal peripheral scotoma	Upper nasal peripheral scotoma
19	Upper nasal peripheral scotoma	Upper arched scotoma

## Discussion

In 2009, the World Health Organization Guideline recommended adding ethambutol throughout the standardized treatment of new cases of active TB in populations with an increased prevalence of resistance to isoniazid, with the goal of reducing the risk of creating strains resistant to multiple drugs. The Brazilian population is included in this at-risk population [4].

The basic TB treatment regimen in Brazil for new cases of pulmonary or extrapulmonary TB with or without HIV and patients over 10 years of age is a combination of fixed-dose tablets of 150 mg of rifampicin, 75 mg of isoniazid, 400 mg of pyrazinamide, and 275 mg of ethambutol for two months and after 150 mg of rifampicin, 75 mg of isoniazid for four months. For children under 10 years of age, the combination of rifampicin, isoniazid, and pyrazinamide remains. The number of tablets administered daily is based on the patient’s weight range, i.e., between 20 and 35 kg two tablets/day, between 36 and 50 kg three pills/day, and over 50 kg four pills/day [2, 4].

Central scotoma is the most common visual field defect [5], the same as our research showed, but bitemporal defects [6] and peripheral field constriction have also been described [7].

The current research showed PSD decreased after treatment compared to first month, therefore campimetry can be a screening test. Considering the learning effect, MD and PSD did not change significantly between months 1 and 2.

Dyschromatopsia is reported as the first symptom of toxic optic neuropathy. Garg et al. observed color vision abnormalities 12.6% of the eyes of 64 patients studied [8]. Some researchers have related a red-green dyschromatopsia, but others have found a predominantly blue-yellow one [9]. Changes in the Ishihara test were observed in the second month of treatment with RHZE, with improvement at post treatment in our study.

Ishihara test is considered the gold standard for the identification of congenital deficiencies for color vision, mainling for the red-green color abnormalities, but it can also be used for acquired deficiencies [10–12]. Because, an expressive percentage of the population has some degree of color vision alteration, the congenital dyschromatosis and changes in the baseline Ishihara test were considered exclusion factors. The Ishihara test was used due to its ease of application, thinking in a primary care context.

Patients treated with ethambutol 25 mg/kg/day had a 5% to 6% incidence of optic neuropathy; patients receiving doses of 15 mg/kg/day had less than a 1% incidence [13, 14]. The current study the mean dose was 18.72 mg/kg/day. The basic TB treatment regimen in Brazil is a combination of fixed-dose tablets, at certain weight ranges, what determines that some patients receive a higher dose per kilogram than others.

Symptoms usually develop between 4 and 12 months of ethambutol use, but they also can develop, although rarely, after a few days of starting therapy [15]. We observed VA decreases after 6 months of treatment. The fundoscopy findings typically are normal, especially in the early stages of the disease. Therefore, studies evaluating the risk factors for development of optic neuropathy due to the use of ethambutol and development of tests to identify the signs of this neuropathy early are important and necessary for clinical practice.

A limitation of this study was the inability to evaluate the use of ethambutol alone.

## Conclusion

The VA and PSD decreased significantly after RHZE treatment. Changes in the Ishihara test were observed in the second month of treatment.

## Data Availability

The datasets used and/or analysed during the current study are available from the corresponding author.

## Abbreviations

MD: Mean deviation
PSD: Pattern standard deviation
RHZE: Rifampicin, isoniazid, pyrimethamine, ethambutol
TB: Tuberculosis
VA: Visual acuity
